# Comparison of first trimester preeclampsia combined screening performances with various approaches in the Indonesian population

**DOI:** 10.1038/s41372-025-02316-y

**Published:** 2025-05-21

**Authors:** Adly Nanda Al Fattah, Muhammad Pradhiki Mahindra, Mirani Ulfa Yusrika, Muhammad Pradhika Mapindra, Felix Firyanto Widjaja, Vania Permata Putri, Shinda Marizni, Sara L. Hillman, Raden Aditya Kusuma

**Affiliations:** 1 Indonesian Prenatal Institute, Jakarta, Indonesia; 2 Kosambi Maternal and Children Center, Jakarta, Indonesia; 3 University College London Elizabeth Garrett Anderson Institute for Women’s Health, London, UK; 4 Harapan Kita National Women and Children Hospital, Jakarta, Indonesia

**Keywords:** Epidemiology, Pre-eclampsia

## Abstract

**Introduction:**

This study aimed to compare Fetal Medicine Foundation (FMF), Indonesian Maternal and Children Health Handbook (MCH-HB), and Indonesian Prenatal Institute (IPI) models for predicting PE.

**Materials/subjects and methods:**

Maternal risk factors, biophysical, and biochemical markers were recorded from women screened for PE at 11–14 gestational weeks. The receiving operator curve (ROC) analysis was used to compare the detection rate (DR) among prediction models.

**Results:**

For all PE at a 10% false-positive rate (FPR), FMF had a DR 62.9%; MCH-HB had a DR 50.0%; IPI had a DR 66.9%. For early-onset PE, at 10% FPR FMF had a DR 80.3%; MCH-HB had a DR 71.4%; IPI had a DR 81.5%. For preterm PE at 10% FPR, FMF had a DR 70.2%; MCH-HB had a DR 38.5%; IPI had a DR 66.9%.

**Discussion:**

IPI algorithm is comparable to FMF and outperforms MCH-HB algorithm for all, early-onset, and preterm PE screening.

## Introduction

Preeclampsia (PE) continues to pose a significant global burden, affecting approximately 2–10% of pregnancies worldwide [1, 2]. The incidence of PE in low middle-income countries (LMIC) is estimated by the World Health Organization (WHO) to be sevenfold greater (1.8–16.7%) than in high-income countries (0.4%) [1–3]. Despite substantial research, PE persists as one of the main contributors to maternal mortality and morbidity globally. It accounts for up to 26% of maternal mortality in low-income countries and 16% in high-income countries [1]. Early PE identification is crucial to minimize maternal and perinatal consequences [4]. In Indonesia, PE serves as the second leading of the maternal mortality with a rate of 26.47% per 100,000 livebirths following postpartum bleeding (38.24% per 100,000 live births) [5]. Numerous studies have been carried out over the last decade to develop a more effective screening tool, reduce the incidence of adverse events, and initiate early aspirin administration for high-risk women [6–9] with first-trimester screening becoming a popular area of investigation. In Indonesia, PE screening before the 20^th^ week of gestation has been made available using a checklist-based method based on the Indonesian Maternal Child Health Handbook (MCH-HB) or “*Buku Kesehatan Ibu dan Anak*” [10]. The MCH-HB algorithm considers potentially harmful pregnancy if two moderate risks or one severe risk are identified, which comprise the following conditions of moderate risks: multiparity with a new partner, assisted pregnancy (in-vitro fertilization/IVF or ovulation drugs), age >35 years, nulliparous, multiparity with pregnancy interval >10 years, family history of PE (mother or sister), obesity (body mass index/BMI > 30 kg/m^2^), mean arterial pressure (MAP) > 90 mmHg, and positive proteinuria results (dipstick urine >1+ on two tests 6 hours apart or immediately quantitative 300 mg/24 h); and high risks: previous pregnancy with PE, gestational diabetes, chronic hypertension, kidney disease, autoimmune disease/systemic lupus erythematosus (SLE), and antiphospholipid syndrome [10]. In contrast, the FMF algorithm combines maternal risk factors with MAP, uterine artery pulsatility index (UtA-PI), placental growth factor (PlGF) serum, and pregnancy-associated plasma protein-A (PAPP-A) [7–9].

Ophthalmic artery Doppler assessment is a feasible and reliable technique for predicting PE in early and late pregnancy [11, 12]. Maternal ophthalmic peak ratio (Oph-PR) is the most established color Doppler index used for ophthalmic artery evaluation [13]. The IPI algorithm integrates maternal risk factors such as nulliparous, pregnancy interval <1 year, assisted pregnancy (IVF or ovulation drugs), previous pregnancy with PE, history of diabetes mellitus, chronic hypertension, family history of PE (mother or sister), age, and BMI, with MAP, UtA-PI, PlGF, and maternal Oph-PR Doppler [14]. Our IPI algorithm is the first-trimester Bayesian survival-time predictive model incorporating ophthalmic artery Doppler and other parameters to predict early-onset and preterm PE [14].

This study evaluated whether the MCH-HB, FMF, or IPI algorithm is superior in predicting PE in the Indonesian population.

## Methods

We performed a prospective cohort study at Harapan Kita National Women and Children Hospital and Kosambi Maternal and Children Centers, Jakarta, Indonesia, from August 2019 to December 2022. Before participation, each individual provided informed consent. This study was conducted according to the institutional research ethics approved by *Yayasan penelitian dan pengembangan obstetrik Indonesia* (reference number 006.ETIK-YPPOI.V.2024). Women with a singleton pregnancy were eligible. Cases involving significant fetal abnormalities or non-viable pregnancies (miscarriages occurring before <24 weeks of gestation) were excluded. Patients underwent first trimester preeclampsia screening between 11 and 14 weeks gestation as per FMF screening Were checked for the Oph-PR as well as assessed using MCH-HB checklists and those screened as high-risk were prescribed low-dose aspirin with the exact dosage determined by the local policy (160 mg/day).

All demographic and medical history details of participants were extracted from electronic medical records. Due to a paucity of record keeping, data regarding proteinuria were unavailable. Maternal MAP was measured using calibrated automated devices (3BTO-A2; Microlife, Taipei, Taiwan), adhering to the recommended 11–14 weeks’ gestation measurement protocol [15]. Gestational age was ascertained based on crown-rump length (CRL) of 45–84 mm. Ultrasound assessments were competently performed by certified sonographers trained by the FMF in Preeclampsia Screening and Doppler ultrasound. UtA-PI was measured transabdominally using color Doppler ultrasound in accordance with the recommended protocol for predicting PE at 11–13 weeks [16]. Additionally, the Oph-PR Doppler was conducted using the protocol outlined in a prior study by Kusuma et al. [14] with either an E8 or P8 Voluson^TM^ machine (GE Healthcare, Milwaukee, WI, USA). Serum PlGF was collected during the visit and measured using the electrochemiluminescence assay method (Cobas E411 analyzer, Roche Diagnostics, USA) [17].

Maternal and pregnancy characteristics such as maternal age (<35 or >35 years old), obesity (BMI < 30 or >30 kg/m^2^), smoking (yes or no), mode of conception (spontaneous and assisted, including ovulation drugs or IVF), parity (nulliparous/parous), pregnancy interval (nulliparous/< 1-year interval or >1-year(s) interval), previous pregnancy with PE (yes or no), previous pregnancy with fetal growth restriction (FGR) (yes or no), previous pregnancy delivery outcome (term birth, preterm birth, or miscarriage), and family history of PE in mother or sister (yes or no). Pregnancy complications included a history of diabetes mellitus (type I or II), chronic hypertension, SLE, antiphospholipid syndrome, and gestational diabetes mellitus (GDM).

Pregnancy outcome data were extracted from delivery room records. PE, was defined as per the International Society for the Study of Hypertension in Pregnancy (ISSHP). It was characterized by gestational hypertension occurring at or beyond the 20^th^ week of gestation accompanied by ≥ 1 of the following new-onset conditions: proteinuria, acute kidney injury, liver involvement, neurological complications, hematological complications, and/or uteroplacental dysfunction [18]. PE was classified based on time of delivery weeks’ gestation and categorized into early-onset PE (delivery with PE < 34 + 0 weeks gestation), preterm PE (delivery with PE < 37 + 0 weeks gestation), and term PE (delivery with PE > 37 weeks gestation) [8].

Sample size calculation was performed according to the primary outcome measures (early PE, preterm PE, and late PE) detected by

FMF-model, as per gold standard. By assuming the statistical power to be 80%, 2-tailed type 1 error rate of 5%, and the proportion of preterm PE identified by FMF model for first trimester screening in Asian population, at approximately 13% [19], the sample size for preeclampsia was determined to be at least 36 women with PE.

Regarding statistical analysis, categorical data were expressed as n (%), and numerical data were reported as mean (standard deviation [SD]). Normality plots were done using Kolmogorov–Smirnov test due to the assumption of large sample size. Maternal and pregnancy characteristics were compared against outcomes through a chi-square test in bivariate analysis, with significance set at a *p*-value of <0.05. The receiving operator curve (ROC) analysis was used to ascertain the area under the curve (AUC) with a 95% confidence interval (CI) and detection rate (DR) of the model in predicting the occurrence of all, early-onset, preterm, and term PE. The Statistical Package for the Social Sciences (SPSS) version 29 for Mac (IBM Corp., Armonk, NY, USA) was utilized for data analysis.

## Results

A total of 1094 women with singleton pregnancies in our cohort underwent the first-trimester screening for PE. Among these, 10 (0.91%) cases necessitated exclusion due to miscarriage, while 47 (4.29%) cases could not have their risk assessed due to missing variables. Consequently, 1037 remaining subjects were suitable for the analysis. Within this cohort, PE developed in 36 (3.47%) women. The incidence of early-onset, preterm, and term PE in our cohort was 0.67% (7/1037), 1.25% (13/1037), and 1.54% (16/1037), respectively. According to Kolmogorov–Smirnov test, the data was abnormally distributed.

Table 1 compares the maternal and pregnancy characteristics against the various outcome groups. Significant variables with higher proportions in the PE group observed in the bivariate analysis included history of previous pregnancy with PE, previous pregnancy with FGR, history of preterm delivery, family history of PE, chronic hypertension, gestational diabetes, and higher MAP. Table 2 displays the mean values of biophysical and biomarker parameters. Compared to the unaffected group, the PE group had older age, greater BMI, lower PlGF level, higher MAP, higher UtA-PI, and higher Oph-PR value.

**Table 1:** Maternal and pregnancy characteristics (*n* = 1037).

Variables		Non-PE		PE								*p*-Value
				All		Early (<34 wks)		Preterm (<37 wks)		Term (>37 wks)		
		*n* = 1001	%	*n* = 36	%	*n* = 7	%	*n* = 13	%	*n* = 16	%	
Demographic profile												
Age (years)	<35	888	88.7	30	83.3	5	71.4	12	92.3	13	81.3	0.320
	>35	113	11.3	6	16.7	2	28.6	1	7.7	3	18.8	
Obesity	No	863	86.2	27	75.0	5	71.4	10	76.9	12	75.0	0.058
	Yes	138	13.8	9	25.0	2	28.6	3	23.1	4	25.0	
Pregnancy characteristics												
Mode of conception	Spontaneous	982	98.1	35	97.2	7	100	13	100	15	93.8	0.577
	Assisted	19	1.9	1	2.8	0	0	0	0	1	6.3	
Parity	Parous	504	50.3	17	47.2	5	71.4	5	38.5	7	43.8	0.522
	Nulliparous	497	49.7	19	52.8	2	28.6	8	61.5	9	56.3	
Pregnancy interval (years)	Nulliparous/<10	974	97.3	35	97.2	7	100	13	100	15	93.8	0.724
	>10	27	2.7	1	2.8	0	0	0	0	1	6.3	
Previous pregnancy with PE^a^	No	969	96.8	26	72.2	3	42.9	12	92.3	11	68.8	**<0.001**
	Yes	32	3.2	10	27.8	4	57.1	1	7.7	5	11.9	
Previous pregnancy with FGR^a^	No	978	97.7	32	88.9	5	71.4	12	92.3	15	93.8	**<0.001**
	Yes	23	2.3	4	11.1	2	28.6	1	7.7	1	6.3	
Previous pregnancy Delivery outcome (*n* = 521 parous)^a^	Term Birth	444	88.1	10	58.8	3	60.0	2	40.0	5	71.4	**<0.001**
	Preterm Birth	41	8.1	7	41.2	2	40.0	3	60.0	2	28.6	
	Miscarriage	19	3.8	0	0	0	0	0	0	0	0	
Family history of PE (Mom/Sister)^a^	No	973	97.2	30	83.3	4	57.1	12	92.3	14	87.5	**<0.001**
	Yes	28	2.8	6	16.7	3	42.9	1	7.7	2	12.5	
Medical history												
Smoking	No	994	99.3	36	100	7	100	13	100	16	100	0.615
	Yes	7	0.7	0	0	0	0	0	0	0	0	
Diabetes Mellitus (Type I or II)	No	996	99.5	35	97.2	7	100	12	92.3	16	100	0.077
	Yes	5	0.5	1	2.8	0	0	1	0.7	0	0	
Chronic HT^a^	No	993	99.2	29	80.6	4	57.1	10	76.9	15	93.8	**<0.001**
	Yes	8	0.8	7	19.4	3	42.9	3	23.1	1	6.3	
SLE	No	999	99.8	36	100	7	100	13	100	16	100	0.788
	Yes	2	0.2	0	0	0	0	0	0	0	0	
APS	No	1000	99.9	36	100	7	100	13	100	16	100	0.850
	Yes	1	0.1	0	0	0	0	0	0	0	0	
Pregnancy outcome												
MAP^a^	<90	729	72.8	11	30.6	1	14.3	5	38.5	5	31.3	**<0.001**
	> 90	272	27.2	25	69.4	6	85.7	8	61.5	11	68.8	
GDM^a^	No	989	98.8	32	88.9	6	85.7	11	84.6	15	93.8	**<0.001**
	Yes	12	1.2	4	11.1	1	14.3	2	15.4	1	6.3	
First trimester screening algorithm												
FMF^a^	Low Risk	880	87.9	13	36.1	1	14.3	5	38.5	7	43.8	**<0.001**
	High Risk	121	12.1	23	63.9	6	85.7	8	61.5	9	56.3	
MCH-HB^a^	Low Risk	690	68.9	9	25.0	0	0	5	38.5	4	25.0	**<0.001**
	High Risk	311	31.1	27	75.0	7	100	8	61.5	12	75.0	
IPI PE < 37 weeks^a^	Low Risk	966	96.5	26	72.2	4	57.1	9	69.2	13	81.3	**<0.001**
	High Risk	35	3.5	10	27.8	3	42.9	4	30.8	3	18.8	

*PE* Preeclampsia, *FGR* Fetal Growth Restriction, *HT* Hypertension, *SLE* Systemic Lupus Erythematosus, *APS* Antiphospholipid Syndrome, *MAP* Mean Arterial Pressure, *GDM* Gestational Diabetes Mellitus, *FMF* Fetal Medicine Foundation, *MCH-HB* Kesehatan Ibu dan Anak, *IPI* Indonesian Prenatal Institute

^a^Chi-square analysis reveals a significant correlation between the unaffected group and the PE group (*p* < 0.05). The bold *p*-Values indicate significant differences among comparisons.

**Table 2:** Biophysical and biomarker measurement values (*n* = 1037).

Variables	Non-PE	PE			
		All	Early (<34 wks)	Preterm (<37 wks)	Term (>37 wks)
	*n* = 1001	*n* = 36	*n* = 7	*n* = 13	*n* = 16
BMI, mean (SD)	24.79 (5.17)	27.84 (6.14)	29.93 (7.30)	26.35 (6.77)	28.13 (5.09)
MAP, mean (SD)	85.36 (10.02)	97.79 (11.00)	103.94 (14.57)	96.22 (9.57)	96.38 (10.12)
PlGF (pg/ml), mean (SD)	57.48 (32.56)	45.49 (19.55)	43.75 (22.95)	42.69 (16.69)	48.52 (20.97)
PlGF (MoM), mean (SD)	1.12 (0.59)	0.99 (0.40)	1.02 (0.50)	0.93 (0.32)	1.02 (0.44)
UtA-PI, mean (SD)	1.64 (0.43)	1.74 (0.47)	1.79 (0.27)	1.76 (0.53)	1.71 (0.51)
Oph, mean (SD)	0.58 (0.15)	0.65 (0.12)	0.72 (0.16)	0.65 (0.13)	0.63 (0.09)

Data are given as mean (standard deviation).

*PE* Preeclampsia, *BMI* Body Mass Index, *PlGF* Placental Growth Factor, *MAP* Mean Arterial Pressure, *UtA-PI* Uterine Artery Pulsatility Index, *Oph-PR* Ophthalmic Artery Peak Ratio.

**Fig. 1:**
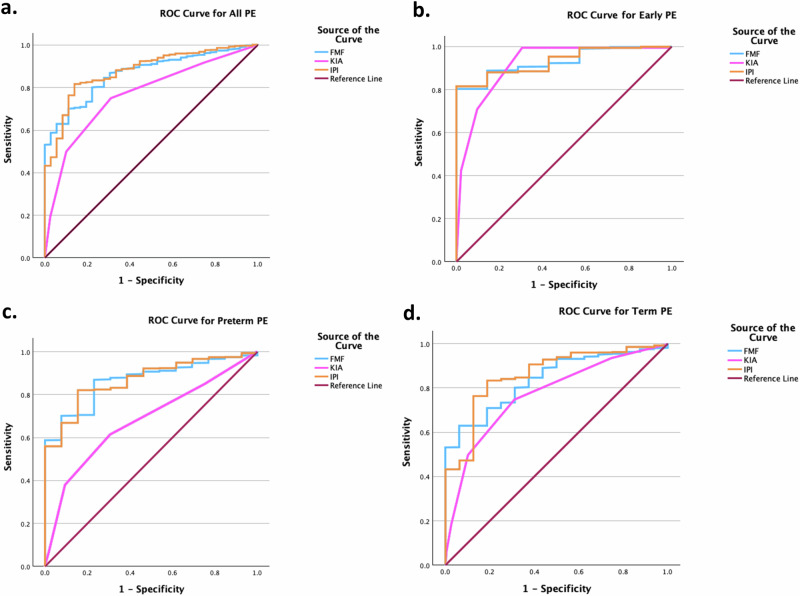
ROC Curve Analysis for preeclampsia prediction models. The receiver operating characteristics (ROC) curve shows the detection rate (DR) of the prediction models using maternal-child health handbook (MCH-HB) or KIA, fetal medicine foundation (FMF), and Indonesian prenatal institute (IPI) for all preeclampsia (PE) (**a**); Early PE (**b**); Preterm PE (**c**); Term PE (**d**).

Table 3 provides an overview of the FMF algorithm’s first-trimester screening performance compared to the MCH-HB and IPI algorithms. At a 10% false-positive rate (FPR), the performance of the algorithms for predicting PE at any gestational age was as follows: FMF had an AUC 0.866 (95% CI 0.822–0.909) and DR 62.9%; MCH-HB had an AUC 0.765 (95% CI 0.677–0.853) and DR 50.0%; IPI had an AUC 0.877 (95% CI 0.830–0.924) and DR 66.9%. For early-onset PE at a 10% FPR, FMF had an AUC 0.930 (95% CI 0.879–0.981) and DR 80.3%; MCH-HB had an AUC 0.916 (95% CI 0.850–0.981) and DR 71.4%; IPI had an AUC 0.931 (95% CI 0.881–0.982) and DR 81.5%. For preterm PE at a 10% FPR, FMF had an AUC 0.866 (0.800–0.932) and DR 70.2%; MCH-HB had an AUC 0.675 (95% CI 0.509–0.841) and DR 38.5%; IPI had an AUC 0.869 (95% CI 0.800–0.938) and DR 66.9%. Visualization of the ROC curves is provided in Fig. 1.

**Table 3:** Summary of receiving operator curve (ROC) Analysis (*n* = 1037).

Outcome		FMF	MCH-HB	IPI
All PE	AUC (95% CI)	0.866 (0.822–0.909)	0.765 (0.677–0.853)	0.877 (0.830–0.924)
	DR at FPR 10%	62.9%	50.0%	66.9%
	DR at FPR 20%	73.5%	62.5%	82.4%
Early PE	AUC (95% CI)	0.930 (0.879–0.981)	0.916 (0.850–0.981)	0.931 (0.881–0.982)
	DR at FPR 10%	80.3%	71.4%	81.5%
	DR at FPR 20%	88.8%	84.8%	88.1%
Preterm PE	AUC (95% CI)	0.866 (0.800–0.932)	0.675 (0.509–0.841)	0.869 (0.800–0.938)
	DR at FPR 10%	70.2%	38.5%	66.9%
	DR at FPR 20%	70.5%	49.5%	82.1%
Term PE	AUC (95% CI)	0.838 (0.766–0.909)	0.772 (0.647–0.898)	0.859 (0.777–0.942)
	DR at FPR 10%	62.9%	50.0%	47.3%
	DR at FPR 20%	70.9%	61.4%	83.2%

*PE* Preeclampsia, *FMF* Fetal Medicine Foundation, *MCH-HB* Kesehatan Ibu dan Anak, *IPI* Indonesian Prenatal Institute, *AUC* Area Under the Curve, *CI* Confidence Interval, *DR* Detection Rate, *FPR* False Positive Rate.

## Discussions

**Fig. 2:**
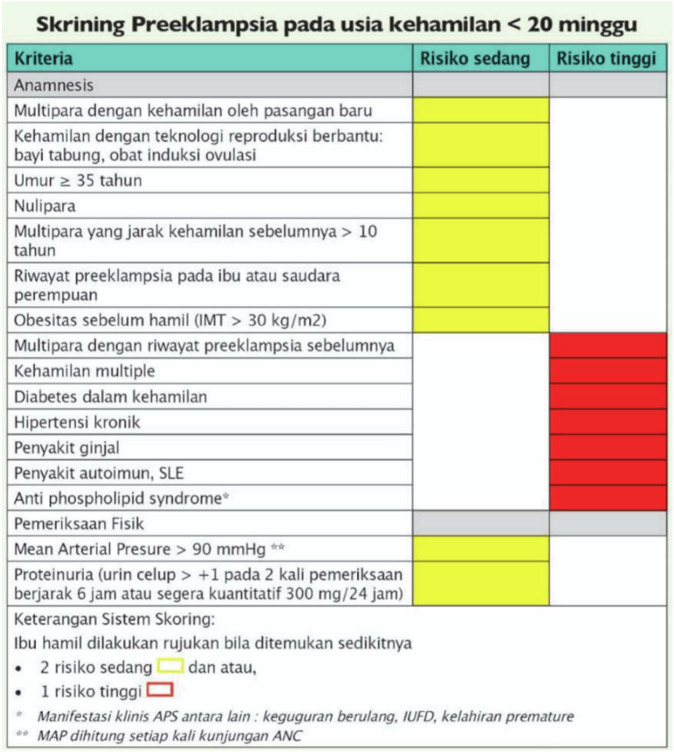
MCH-HB checklist and scoring system for PE prediction.

This study validated the performance of first-trimester screening for PE employing various approaches in Indonesia, including the triple tests from FMF, the Indonesian-based MCH-HB (Fig. 2), and our institutional-based IPI algorithms. To the best of our knowledge, this is the first study ever to examine the performance of the MCH-HB algorithm, which stands as the most frequently implemented screening algorithm <20 weeks of gestation for PE in LMIC countries including Indonesia, explicitly issued by the Indonesian Association of Obstetrics and Gynecology in 2020 [10]. In addition, we integrated the utilization of Oph-PR along with the combined variables in our first-trimester Bayesian survival-time predictive model, IPI algorithm [14].

The first-trimester PE combined screening with the FMF algorithm has been extensively used and validated across multiple nations and studies [6, 7, 20–23]. The vast majority of the validation studies have consistently upheld comparable predictive performance akin to the original research [9]. Likewise, our findings resonated with satisfaction. The screening performance of the FMF algorithm within our cohort overall exhibited good accuracy (AUC > 0.800), with the DRs at a 10% FPR standing at 62.9% and 70.2% for PE at any gestational age (AUC 0.866 [95% CI 0.822–0.909]) and <37 weeks of gestation (AUC 0.866 [0.800–0.932]), respectively. Of particular note, the model accurately predicted early-onset PE with AUC 0.930 (95% CI 0.879–0.981) and DR of 80.3% at a 10% FPR.

A previous FMF triple test study with a multiethnicity population by Tan et al. suggested the DRs at 10% FPR for early-onset PE, preterm PE, and term PE were 90.0%, 81.7%, and 42.6%, respectively [6]. While the AUC values have a resemblance to our findings, indicating they are a good predictor of early-onset and preterm PE, the DRs in our investigation were inferior compared to the previous study. However, a large East Asian population study showed that although the accuracy was suitable for their demographic, the DR was slightly lower compared to the study with the Caucasian population [9, 22, 24].

The respective DRs at a 10% FPR in screening with the IPI algorithm were 66.9%, 81.5%, and 66.9% for all, early-onset, and preterm PE. Similar to the FMF algorithm, the IPI algorithm overall showed good accuracy in predicting all PE (AUC 0.877 [95% CI 0.830–0.924]) and preterm PE (AUC 0.869 [95% CI 0.800–0.938]) and excellent accuracy in predicting early-onset PE (AUC 0.931 [95% CI 0.881–0.982]). Thus, both algorithms are comparable. ROC analysis produced no improvement compared to our earlier study using the identical technique [14]. With a 10% FPR, the model exhibited excellent accuracy in predicting early-onset PE (DR 100%, AUC 0.981 [95% CI 0.965–0.998]) and preterm PE (DR 71%, AUC 0.919 [0.875–0.963]) [14].

The ophthalmic artery was reported to be equally effective with the uterine artery, the most investigated biophysical marker, in predicting early-onset PE in the first trimester; nevertheless, their combined use fails to augment the DR [11]. In contrast, Gana et al., in their study conducted during 11–13 weeks gestation, illustrated that incorporating the second-to-first peak systolic velocity (PSV) ratio of maternal ophthalmic to maternal factors, MAP, UtA-PI, and PlGF enhanced the prediction accuracy of preterm PE (DR 74.6–76.7% at FPR 10%), with no discernible improvement in term PE prediction with any combination of biomarkers [25].

In screening based on the MCH-HB algorithm, the DRs of PE at any gestational age, <34, and <37 weeks of gestation at a 10% FPR were 50.0%, 71.4%, and 38.5%, respectively. Despite the model exhibited fair accuracy in predicting all PE (AUC 0.765 [95% CI 0.677–0.853]) and poor accuracy in predicting preterm PE (AUC 0.675 [95% CI 0.509–0.841]), the MCH-HB algorithm had excellent accuracy in predicting early-onset PE (AUC 0.916 [95% CI 0.850–0.981]). This finding may warrant bias because no data on proteinuria was provided, which was one of the values to be included in the screening.

The MCH-HB algorithm emerges as a first-trimester PE screening approach that combines maternal characteristics, history, BMI, MAP, and proteinuria measurement [10]. Due to record-keeping limitations, proteinuria was not included in our study. Previous research has extensively evaluated the accuracy of proteinuria in predicting preeclampsia (PE) using methods such as urine dipstick tests, 24-h urine collection, and the protein-to-creatinine ratio [26]. A systematic review of 16 studies revealed variability in the proteinuria thresholds employed, indicating a lack of standardization [27]. Another review identified the optimal threshold for detecting proteinuria as 0.30–0.35, with sensitivity and specificity estimates exceeding 75% [28]. However, the accuracy and appropriate clinical thresholds remain inconsistent, limiting widespread application without further standardization. In Indonesia, proteinuria screening predominantly utilizes urine dipstick tests, which are subjectively interpreted and can yield positive results at +1, +2, or +3. High proteinuria in early pregnancy may indicate previous glomerular damage, necessitating further evaluation for new-onset proteinuria [29]. Additionally, studies suggest that first-trimester proteinuria screening for PE is most effective when combined with other maternal, biophysical, and biochemical markers [30].

The MCH-HB algorithm method is very suitable for use in low-middle resource settings, where Indonesia is an archipelagic country with disparate access to healthcare facilities. In low- and middle-income countries (LMICs), tests such as PLGF and UtA-PI are not readily accessible in primary healthcare settings [9]. Tan et al., in their research, showcased that a combination of maternal factors alone or with a combination of MAP can diagnose preterm PE at DR 44.8% and 50.5%, respectively [24]. Therefore, the International Federation of Gynecology and Obstetrics (FIGO) suggests that while baseline testing includes maternal variables with MAP, modifications of the first trimester combined test can be considered in LMICs with limited resources [8].

Our findings unveil the commendable predictive precision of the FMF and IPI algorithms in differentiating PE across multiple gestational ages. Moreover, rooted in Indonesian practice, the MCH-HB algorithm displayed remarkable predictive efficacy, particularly in early-onset PE detection. Identifying pregnancies at high risk of developing PE earlier is vital to reduce the occurrence of these complications through pharmacological intervention, such as medication with low-dose aspirin in the first trimester [2, 31]. Administration of low-dose aspirin earlier in pregnancy to populations at heightened risk is beneficial in preventing preterm rather than term PE [4, 9]. Previous research stated that approximately 10% of the pregnant women population would receive low-dose aspirin, and this population would include 75% of those who would develop preterm PE if the high-risk group was chosen based on multimodal tests rather than only 39% if the choice was made based on maternal factors alone [7].

Notably, while the combined utilization of ophthalmic and uterine artery parameters showed promise in enhancing the prediction of early-onset PE, the overall effectiveness remained comparable to utilizing maternal parameters and MAP alone despite differences in the DR. This underscores the importance of refining screening algorithms to optimize predictive accuracy while considering resource constraints, particularly in low-middle-income settings. Our findings contribute to the evolving PE screening methodologies, providing valuable insights for clinicians and policymakers in tailoring screening protocols. FIGO encourages all nations and its member associations to ensure regular first-trimester evaluation at all maternal health facilities, including risk assessment and resource-appropriate testing for PE [8, 9]. This enables early intervention to mitigate the maternal and perinatal sequelae associated with PE. Future research may explore novel biomarkers and refined approaches to enhance the precision of PE prediction, particularly in limited resource settings.

This study was meticulously conducted to advance our understanding of first-trimester PE combined screening, particularly in the context of the Indonesian population. Its strengths lie in its pioneering effort employing a multi-methodological approach to investigate the performance of various screening algorithms in a relatively large sample of pregnant women in Indonesia, including the novel examination of the MCH-HB algorithm, which fills a crucial gap in the literature and provides clinicians and policymakers with valuable insights for optimizing PE screening protocols. Another commendable aspect is that screening is carried out by certified FMF sonographers, and data were audited regularly. However, some notable limitations should be addressed. This study, conducted in the capital city of Jakarta, raises concerns about its applicability to the larger Indonesian population. Cultural, genetic, and socioeconomic factors unique to this population may influence the performance of screening algorithms differently in other settings. The absence of complete data for all variables, such as proteinuria measurements, may impact the accuracy and generalizability of the findings. Moreover, considerations regarding the feasibility and cost-effectiveness of different screening algorithms, particularly in resource-limited settings, could further enhance the study’s relevance.

This comprehensive investigation delved into the efficacy of various first-trimester screening algorithms for predicting preeclampsia (PE) in an Indonesian population. Our results showed good predictive power for early-onset and preterm PE but less for term PE. The IPI algorithm, which integrates ophthalmic artery Doppler PR with maternal factors, biophysical, and biochemical markers, is comparable to the FMF and outperforms the MCH-HB algorithm for all, early-onset, and preterm PE screening during 11–14 weeks gestation in the Indonesian population.

## Data Availability

The data of our study participants has been anonymised and not distributed to other parties.
